# Difficult endotracheal intubation due to a large epiglottic cyst: A case report

**DOI:** 10.1097/MD.0000000000034026

**Published:** 2023-06-16

**Authors:** Ran Zhang, Xian Jiang, Jianguo Feng

**Affiliations:** a Department of Anesthesiology, the Affiliated Hospital of Southwest Medical University, Luzhou, Sichuan Province, China; b Anesthesiology and Critical Care Medicine Key Laboratory of Luzhou, Department of Anesthesiology, Southwest Medical University, Luzhou, Sichuan Province, China; c Department of Anesthesiology, Luzhou People’s Hospital, Luzhou, Sichuan Province, China.

**Keywords:** anesthesia management, case report, difficult airway, endotracheal intubation, epiglottic cyst

## Abstract

An epiglottic cyst is a type of benign tumor that is formed due to the obstruction of the mucinous duct and the retention of glandular secretion. In such cases, the glottis is not visible as it is covered by the enlarged epiglottic cyst. When conventional anesthesia is administered in such patients, they might have difficulty ventilation since the epiglottic cyst can easily form a flap and move with external pressure changes and can cause the blockage of the glottis due to the loss of consciousness and the relaxation of the throat muscles of the patient. If endotracheal intubation is not initiated and effective ventilation is not established, the patient may suffer from hypoxia and other accidents.

**Patient concerns:**

A 48-year-old male presented to the otolaryngology department with a foreign body sensation in the throat.

**Diagnoses:**

A large epiglottic cyst was diagnosed.

**Interventions:**

The patient was planned to undergo epiglottis cystectomy under general anesthesia. After induction of anesthesia, the cyst severely covered the glottis and made endotracheal intubation difficult. The anesthesiologist rapidly adjusted the position of the laryngeal lens; thus, the endotracheal intubation was successful under the visual laryngoscope.

**Outcomes:**

The endotracheal intubation was successful under the visual laryngoscope and the operation went well.

**Lessons:**

Patients with epiglottic cysts are more likely to have difficult airways after induction of anesthesia. Anesthesiologists should take preoperative airway assessment seriously, efficiently handle difficult airway and intubation failure, and make quick and correct choices to ensure patient safety.

## 1. Introduction

An epiglottic cyst is a type of benign tumor that is formed due to the obstruction of the mucinous duct and the retention of glandular secretion; it is usually found in the lingual plane of the epiglottis and can occur in patients of all ages.^[1–3]^ Adult epiglottic cysts can be asymptomatic or show mild symptoms. With the growth of the tumor, patients can feel foreign body sensation, pharyngeal pain, a sense of obstruction, and even dyspnea; however, airway obstruction is a rare symptom.^[4]^ The most effective treatment for epiglottic cysts is surgical resection. For patients with epiglottic cysts, even if the clinical symptoms are mild, the perioperative period should be carefully planned to avoid obvious airway obstruction that may be noted immediately after the use of sedation and other anesthesia-induction drugs, leading to difficult airways.^[5,6]^

Herein, we report the case of a male patient with an epiglottic cyst who underwent difficult tracheal intubation due to the site and size of the cyst. Our findings suggest that before managing patients undergoing throat surgery, it is important to carefully assess the site and size of the lesion, check whether it is complicated with a difficult airway, and accordingly select the visual equipment for anesthesia induction.

## 2. Case report

**Figure 1.**
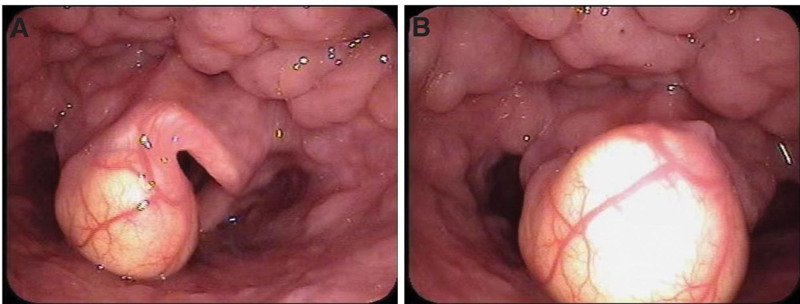
Preoperative images of fiberoptic laryngoscopy.

A 48-year-old male presented to the otolaryngology department with a foreign body sensation in the throat and reported no difficulty in speaking, dyspnea, swallowing obstruction, and other symptoms. Fiberoptic laryngoscopy revealed chronic hyperemia of the pharyngeal mucosa; some lymphatic follicular hyperplasia on the posterior pharyngeal wall; a yellowish, smooth cystic neo-organism on the left side of the free epiglottis margin; symmetrical vocal cords on both sides; good movement and closure (Fig. 1). Based on all these findings, the diagnosis of the epiglottic cyst was formed, and mass resection under a supportive laryngoscope was planned. The operation time was expected to be 20 minutes.

At the preoperative visit, the general condition of the patient was acceptable. Although the patient denied hoarseness, difficulty breathing, and snoring during sleep and although the Mallampati scores, temporomandibular joint movement, neck movement, and thyromental distance were normal and since the epiglottic cyst was large and located in the middle of the free epiglottis margin, exposure of the glottis was difficult, leading to difficulty in endotracheal intubation. A visual laryngoscope was arranged in advance to facilitate observation and improve the success rate of intubation, a laryngeal mask was kept as a backup in case of emergency respiratory maintenance, and otolaryngologists were informed in advance to be prepared for tracheostomy.

After the patient entered the operating room, electrocardiogram monitoring was performed. To increase the oxygen reserve in the patient body and provide rescue time for the possible difficult airway, preoxygenation was performed immediately. Sufentanil (1–2 µg/kg) and propofol (3–5 mg/kg) were administered. Artificial ventilation was provided with a mask; then, succinylcholine (1.5–2 mg/kg) was administered, and endotracheal intubation was performed under a visual laryngoscope. However, after we picked up the epiglottis, we found that the epiglottic cyst was still hanging from the glottis due to its weight, and there was no space for endotracheal intubation. To avoid causing any damage to the cyst, blood vessels, and surrounding mucosa, we did not continue to try endotracheal intubation from just below the cyst. The position of the laryngeal lens was then adjusted and the endotracheal tube was inserted from the side of the cyst into the glottis. The patient vital signs were stable throughout the process.

**Figure 2.**
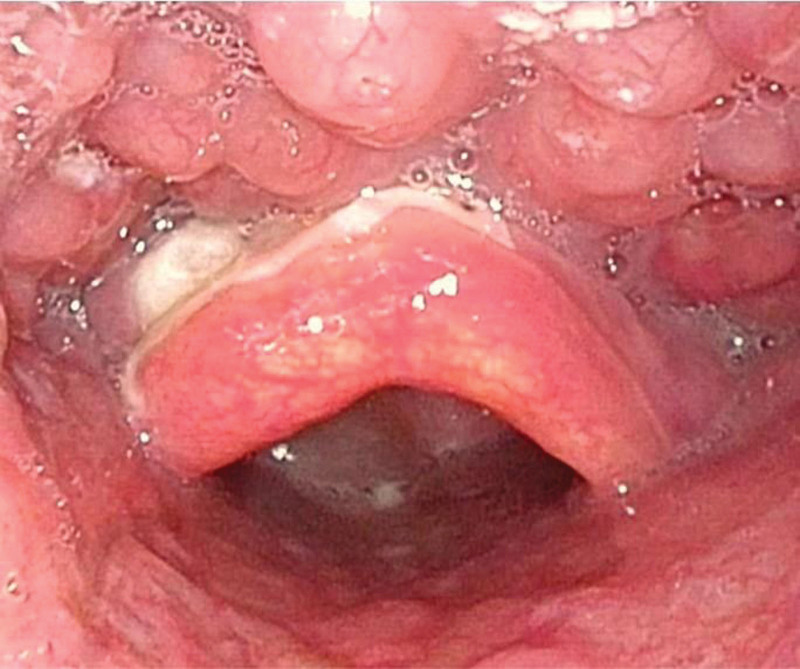
Postoperative images of fiberoptic laryngoscopy after surgery.

The surgery was uneventful and lasted for approximately 30 minutes. The endotracheal tube was removed when the patient was fully awake. The patient did not complain of any particular discomfort the next day. The second fiberoptic laryngoscopy revealed no significant hyperemia and swelling of the epiglottis and showed significant bilateral vocal cord hyperemia with good movement and closure (Fig. 2).

## 3. Discussion

In patients with an epiglottic cyst, the glottis is not visible as it is covered by the enlarged epiglottic cyst. When conventional anesthesia is administered in such patients, they might have difficulty breathing since the epiglottic cyst can easily form a flap and move with external pressure changes and can cause the blockage of the glottis due to the loss of consciousness and the relaxation of the throat muscles of the patient. If endotracheal intubation is not initiated and effective ventilation is not established, the patient may suffer from hypoxia and other accidents. Therefore, the anesthetic treatment of patients with epiglottic cysts has great specificities.^[3]^

In patients with a clear preoperative diagnosis, those with a small cyst, and those who have no obvious symptoms, conventional anesthesia induction can be used. Short-term muscle relaxants, such as succinylcholine, can be used. If intubation fails, awake intubation can be performed after the patient spontaneous breathing resumes. In case the cyst is large and symptomatic, preoperative sedation should be carefully selected to avoid airway obstruction.^[7,8]^ High concentrations of sevoflurane can be an efficient and safe method for the induction of anesthesia and can be used for endotracheal intubation in patients with large epiglottic cysts.^[9]^ Anticholinergics can be used to suppress oral secretions, and dexamethasone can be used to reduce glottal edema. If the cyst is particularly large, it significantly hinders endotracheal intubation, which can easily lead to cyst rupture and aspiration of contents. In such cases, the cyst can be punctured with a fine needle under local anesthesia; then, the cyst fluid can be aspirated to decrease the size and tension of the cyst, thus, endotracheal intubation can be allowed.^[10]^ However, laryngeal masks should be used carefully. Kariya et al reported a case of difficult airway management in a patient with an asymptomatic and undiagnosed epiglottic cyst. The patient had no difficulty in mask ventilation after the induction of general anesthesia but had difficulty ventilation after laryngeal mask intubation because the laryngeal mask pushed the cyst into the laryngeal inlet.^[5]^

Airway control should always be the top priority when inducing anesthesia. To ensure normal ventilation in the patient, management at all stages of the perioperative period should be strengthened. Comprehensive preoperative airway assessment improves the predictability of asymptomatic epiglottic cysts and decreases the incidence of urgent difficult airways. In the case of an urgent and difficult airway, noninvasive methods are preferred; however, tracheostomy should be performed, if required. To avoid adverse events, the airway should be closely and carefully observed and managed postoperatively.^[11]^

## 4. Conclusion

Patients with epiglottic cysts are more likely to have difficult airways after induction of anesthesia. Anesthesiologists should take preoperative airway assessment seriously, efficiently handle difficult airway and intubation failure, and make quick and correct choices to ensure patient safety.

## Author contributions

**Writing – original draft:** Ran Zhang.

Writing – review & editing: Xian Jiang, Jianguo Feng.
